# Efficiency Comparison of a Novel E2 Subunit Vaccine and a Classic C-Strain Vaccine against Classical Swine Fever

**DOI:** 10.3390/vetsci8080148

**Published:** 2021-07-29

**Authors:** Pei Zhou, Junming Huang, Yanchao Li, Hui Chen, Yidan Wu, Xueying Fu, Xiangqi Hao, Qi Li, Rongyu Zeng, Guihong Zhang

**Affiliations:** 1 Guangdong Provincial Key Laboratory of Comprehensive Prevention and Control for Severe Clinical Animal Diseases, College of Veterinary Medicine, South China Agricultural University, Guangzhou 510642, China; zhoupei@scau.edu.cn (P.Z.); vzhoupei@163.com (J.H.); liyanchao@stu.scau.edu.cn (Y.L.); chan.scau.edu.cn@stu.scau.edu.cn (H.C.); wuyidan@stu.scau.edu.cn (Y.W.); 20193073022@stu.scau.edu.cn (X.F.); haoxiangqi@stu.scau.edu.cn (X.H.); haoxiangqi0829@163.com (Q.L.); 2 Tecon Biology Co., Ltd., Urumqi 830000, China; rongyuzeng2011@163.com; 3 Key Laboratory of Zoonosis Prevention and Control of Guangdong Province, College of Veterinary Medicine, South China Agricultural University, Guangzhou 510642, China; 4 Guangdong Laboratory for Lingnan Modern Agriculture, Guangzhou 510642, China

**Keywords:** pigs, classical swine fever, C-strain vaccine, E2 subunit vaccine

## Abstract

Classical swine fever (CSF) is one of the most important viral diseases in swine, causing severe economic losses in the swine industry. In China, CSF is one of the key diseases that needs to be controlled; the government has implemented control measures, and vaccination with C-strain vaccines (C-vacs) has been compulsory since the 1950s. C-vacs do not allow the differentiation of field virus-infected and vaccinated animals (DIVA). In 2012, China proposed a goal of eradicating CSF. Additionally, a baculovirus-expressed E2 subunit vaccine (E2-vac) was licensed in 2018. However, the C-vac and E2-vac characteristics have not been compared. Here, we demonstrate that both the C-vac and E2-vac provide complete protection against CSF in pigs. The E2-vac allows DIVA, and the E2 antibody responses of stimulated pigs are developed earlier and are stronger than the C-vac antibody responses. Therefore, the E2-vac is a new candidate licensed vaccine to completely eradicate CSF on pig farms.

## 1. Introduction

Classical swine fever (CSF) is one of the most important viral diseases in swine, including wild boar. CSF causes severe economic losses in the swine industry worldwide and is a disease listed by the World Organisation for Animal Health (OIE) [1]. CSF is caused by the classical swine fever virus (CSFV), a member of the Pestivirus genus within the Flaviviridae family [1]. Other members of this genus are bovine viral diarrhea virus (BVDV)-1, BVDV-2, and border disease virus (BDV). The CSFV genome consists of a positive single-stranded RNA genome that is approximately 12.3 kb in length and encodes one polyprotein. The translated polyprotein is processed by viral and cellular proteases to form 12 mature proteins, four of which are structural (core protein (C) and envelope glycoproteins E0, E1, and E2) and eight of which are nonstructural (Npro, p7, NS2, NS3, NS4A, NS4B, NS5A, and NS5B) [2]. Among these proteins, E2 is a multi-functional glycoprotein that plays roles in viral attachment, viral replication and host immunoreactions [3,4,5,6]. Additionally, the E2 protein is the major protective antigen inducing neutralizing antibody in the host [7], and has been selected as the most effective immunogen for development of subunit vaccines against CSFV [7,8,9,10].

CSF is distributed nearly worldwide, with subgenotypes 1.1, 1.3, 1.4, 2.1, 2.2, 2.3, 3.2, and 3.4 [11]. Governments have implemented control measures with a nonvaccination, stamping-out policy or a prophylactic vaccination strategy. Currently, the disease is at least sporadically present in several countries in South and Central America, Eastern Europe, and Asia [11]. Nevertheless, after the implementation of strict control measures, several countries, such as the U.S., have succeeded in eradicating CSF [12]. In China, despite the implementation of prophylactic vaccination control measures, CSF is still endemic in many regions. The virus is characterized by high strain variability, primarily in the subgenotypes 1.1, 2.1, 2.2, and 2.3 [13,14,15,16]. Therefore, the complete eradication of CSF in China remains a challenging task using existing measures. China is the largest pork producer in the world, with 441.59 million pigs being slaughtered in 2017 [17]. A modified live virus (MLV) vaccine derived from the C-strain, the administration of which is mandatory, is used to establish protective immunity in naïve pig populations and prevent CSF outbreaks. The main drawback of MLV vaccines, including the C-strain vaccine, is the lack of a serological marker that allows the differentiation of field virus-infected and vaccinated animals (DIVA) [18]. Thus, several E2 subunit marker (DIVA) vaccines have been evaluated worldwide [9,19,20,21,22]. In 2012, China established a goal of eradicating CSF. Several marker vaccines have also been evaluated, including a chimeric adenovirus/alphavirus vector-based vaccine [23,24,25] and a yeast/baculovirus-expressed E2 subunit vaccine [26,27]. Additionally, a baculovirus-expressed E2 subunit vaccine (Rb-03, Tianwenjing) was licensed in 2018. Therefore, two types of CSF vaccines are available, the C-strain vaccine (C-vac) and the E2 subunit vaccine (E2-vac), and Chinese swine farmers must choose which vaccine to use. A study has shown that this commercial E2 subunit vaccine provides full protection to pigs against lethal challenge with CSFV. In this study, we evaluated the protective efficacies and compared different antibodies of the two CSF vaccines, providing important data for farmers to make informed choices regarding which swine vaccine to use.

## 2. Materials and Methods

### 2.1. Virus and Vaccines

The CSFV strain GD11 (2.1 subgenotype) that was isolated from Guangdong Province was used for the challenge experiment. CSFVs belonging to genotype 2.1 are dominant strains in the field and thus we chose the GD11 isolate of this category as a challenge virus. PK-15 cells were cultured in Dulbecco’s modified Eagle’s medium (DMEM) containing 10% fetal bovine serum (FBS; Gibco) and streptomycin at 37 °C in a 5% CO_2_ atmosphere. CSFV titers were determined using log10 dilutions that were added to cells in quadruplicate. Viral titers were calculated as the 50% tissue culture infectious dose (TCID50) per mL. C-vac was obtained from WINSUN Biology Co., Ltd., Guangzhou, China, and E2-vac was obtained from Tecon Biology Co., Ltd., Urumqi, China (Lot 2018009, expiration date 10 November 2019). The E2 sequence in E2-vac is basically copied from the C-strain E2 gene (1.1 genotype) as C-strain has been shown to confer protection against all genotypes of CSFV strains isolated from China including 1.1 and 2.1 (a–j).

### 2.2. Pigs

In total, 30 weaning female pigs (five weeks old) were used for the animal experiments. The piglets were provided by Qingyuan Pig Farm in Guangdong Province. The piglets were tested and shown to be free of antigens from CSFV, BVDV, porcine reproductive and respiratory syndrome virus (PRRSV), porcine pseudorabies virus (PRV) (IDEXX Labs Inc., Westbrook, ME, USA), porcine circovirus type 2 (PCV2) (Biochek, Reeuwijk, The Netherlands), and porcine parvovirus (PPV) ((Laboratoire Service International, Lissieu, France) by ELISA; they also tested negative for antibodies against CSFV and BVDV (IDEXX Labs Inc., Westbrook, ME, USA). All animal studies were conducted in accordance with the guidelines approved by the Animal Care and Use Committee of South China Agricultural University.

### 2.3. Animal Experiment

**Figure 1:**
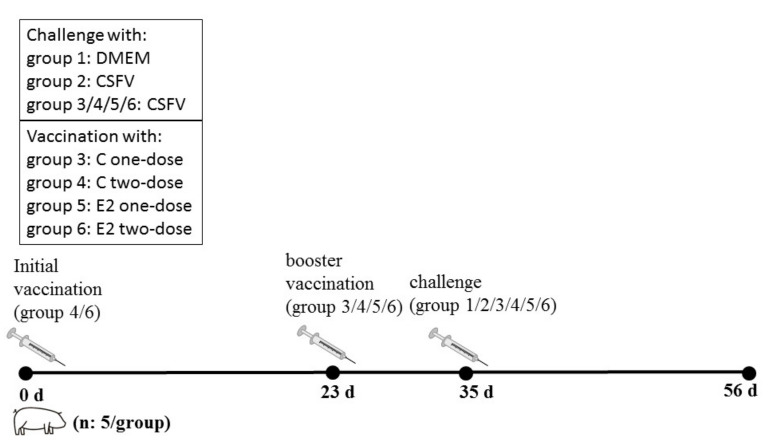
Diagram of the animal experimental design. Piglets received an initial intramuscular injection that was defined as occurring at 0 days post vaccination (dpv).

The animal experiment was conducted by veterinarians who have special training in animal care or handling. The procedure for the animal experiment is presented in Figure 1. The piglets were randomly divided into six groups (5/group). The piglets received an initial intramuscular injection (2 mL each as per the manufacturer’s instructions) that was defined as occurring at 0 days post vaccination (dpv). The blank control group (Group 1: DMEM) received no immunization and no challenge. The infection control group (Group 2: CSFV) received no immunization. Group 3 (C-vac one dose) and Group 5 (E2-vac one dose) pigs were intramuscularly injected with the relevant vaccines (2 mL each as per the manufacturer’s instructions) at 23 dpv. Group 4 (C-vac two doses) and Group 6 (E2-vac two doses) pigs were intramuscularly injected with the relevant vaccines (2 mL each as per the manufacturer’s instructions) at 0 dpv and received a booster immunization at 23 dpv. At 35 dpv, pigs in all groups except Group 1 were challenged with 105 TCID50 GD11 in a 1 mL volume.

Clinical symptoms, including animal health and behavior, were observed daily throughout the study, and rectal temperatures were recorded daily before feeding. Blood samples and oropharyngeal swabs were collected at 0, 1, 2, 5, 7, 9, 12, 16, 20, 23, 28, 30, 35, 37, 40, 42, 44, 47, 51, 55, and 56 dpv for the determination of viral load. The viral load was quantified by real-time reverse transcription polymerase chain reaction (RT-qPCR) using a SYBR Green kit (SYBR Premix Ex Taq, Takara, Japan) and the following primers: CSF-qPCR-F: GCAGAAGCCCACCTCGAGAT and CSF-qPCR-R: TACACCGGTTCCTCCACTCC. The qPCR standard curve was as follows: y = −3.585lyX + 38.845. The serum samples were collected for antibody detection by ELISA (E2 antibody: IDEXX Labs Inc., Westbrook, ME, USA; E0 antibody: Qiagen Labs Inc., Hilden, Germany). In order to minimize animal suffering and distress, animals were euthanized by intravenous injection of pentobarbital within an hour while animals were determined to be moribund (as indicated by increased respiratory rate and inability to ambulate), and all remaining pigs were euthanized at 56 dpv.

### 2.4. Statistical Analysis

ANOVA was used for the analysis of fixed effects on different traits using GraphPad Prism 8 (Prism 5 for Windows, Version 8.01, GraphPad Software, Inc. La Jolla, San Diego, CA, USA).

## 3. Results

### 3.1. Both the C-Vac and the E2-Vac Provided Complete Protection against CSF in Pigs

**Figure 2:**
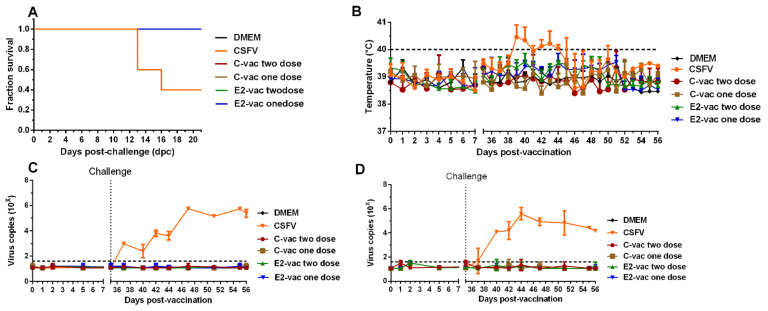
The survival curves, body temperatures, viremia, and virus shedding of the experimental pigs. (**A**) Survival rate, the lines of dates are covered by the lines of DMEM and CSFV data. (**B**) Body temperature. (**C**) Viral copies in oropharyngeal swab samples. (**D**) Viral copies in the blood. The dotted line represents the minimum detection threshold. DMEM: the blank control group that received no immunization and no challenge. CSFV: the infection control group that received no immunization. C-vac one dose and E2-vac one dose: pigs were intramuscularly injected with the relevant vaccines at 23 dpv. C-vac two doses and E2-vac two doses: pigs were intramuscularly injected with the relevant vaccines at 0 dpv and received a booster immunization at 23 dpv. At 35 dpv, pigs in all groups except DMEM group were challenged with ASFV.

In the infection control group, two piglets died at 13 days post challenge (dpc), and one piglet died at 16 dpc (Figure 2A); the remaining two piglets were euthanized due to severe clinical symptoms, including fever (Figure 2B), anorexia, labored breathing, and astasia at 56 dpv. All vaccinated animals from the C-vac and E2-vac groups and the blank control group survived and were healthy (Figure 2A,B). The viral loads were detected and quantified by qPCR. The number of viral copies in the blood (Figure 2C) and oropharyngeal swabs (Figure 2D) were detected from day 37 (2 days post challenge) until the death of the pigs in the infection control group. No virus was detected in pigs in the vaccination groups. Additionally, the viral loads were reevaluated using isolation in PK-15 cells, and these results were consistent with those of the qPCR assays (data not shown). These results suggested that GD11 is fatal for pigs and could cause viremia and virus shedding from the oropharynx, and both C-vac and E2-vac provided complete protection against CSF in the pigs.

### 3.2. C-Vac Stimulated Different Antibodies, and E2-Vac Stimulated Only the E2-Specific Antibody

Antibodies play an important role in protective immunity. As an MLV vaccine, C-vac stimulates the production of antibodies against the whole virus particle. However, the envelope glycoprotein E2 is the only target in the E2-vac and therefore stimulates the production of only the E2-specific antibody. No antibody responses were observed in pigs in the infection control and blank control groups according to the results of the E2 and E0 antibody detection tests (Figure S1).

**Figure 3:**
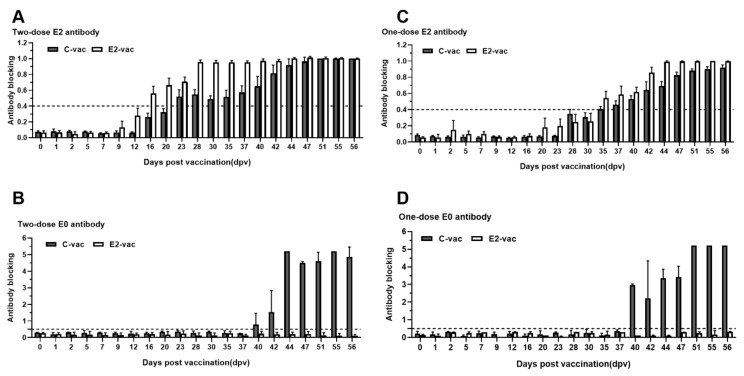
Antibody responses in pigs in the two-dose (**A**,**B**) and one-dose (**C**,**D**) vaccination groups according to the blocking ELISAs. Gray columns represent the ELISA blocking rate of the C-vac groups, and white columns represent the ELISA blocking rate of the E2-vac groups. The dotted line represents a positive threshold: E2 > 0.4, E0 > 0.5, NS3 > 0.25.

In the C-vac two-dose group, one pig first produced E2 antibodies at 20 dpv, and all animals produced E2 antibodies starting at 23 dpv (Table 1). On average, the pigs tested positive for the E2 antibody at 23 dpv (Figure 3A). The antibody level subsequently gradually increased and reached a 100% ELISA blocking rate at 51 dpv (Figure 3A). Two pigs first produced the E0 antibody at 40 dpv, and all animals produced the E0 antibody starting at 42 dpv (Table 1). On average, the pigs tested positive for the E0 antibody at 40 dpv (Figure 3B). Subsequently, the antibody level increased dramatically and reached a 100% ELISA blocking rate at 44 dpv (Figure 3B). In the C-vac one-dose group, four pigs first tested positive for the E2 antibody at 35 dpv, and all animals tested positive at 37 dpv (Table 1). On average, the pigs tested positive for the E2 antibody at 35 dpv (Figure 3C), after which the antibody level increased gradually but did not reach a 100% ELISA blocking rate (Figure 3C). All five pigs had a 100% ELISA blocking rate for the E0 antibody at 40 dpv (Table 1, Figure 3D).

In the E2-vac two-dose group, one pig tested positive for the E2 antibody at 12 dpv, and all animals tested positive at 16 dpv (Table 1, Figure 3A). Subsequently, the antibody level increased dramatically and reached an approximately 100% ELISA blocking rate at 28 dpv (Figure 3A). In the E2-vac one-dose group, one pig tested positive for the E2 antibody at 30 dpv, and all animals tested positive at 35 dpv (Table 1, Figure 3C). The antibody level subsequently increased dramatically and reached an approximately 100% ELISA blocking rate at 44 dpv (Figure 3C). No E0 antibodies were detected in any of the E2-vac groups (Figure 3B,D).

## 4. Discussion

CSF causes severe economic losses in the swine industry in China. Therefore, it is one of the key diseases that needs to be controlled, and the government has implemented control measures, including compulsory vaccination with C-vacs, for decades. The C-vac was developed in China in 1956. The vaccine was shown to be attenuated in rabbits and is generally considered safe and effective [16,28,29]. To date, among a series of CSF vaccines that are in development, an E2-vac has been licensed in China, which increases the options available to pig farmers but makes the choice more difficult.

Previously, a study showed that this commercial E2 subunit vaccine provides full protection to pigs against lethal challenge with different strains of CSFV genotype 2 [30]. Our study showed consistent results that both the C-vac and the E2-vac provide complete protection against CSF in pigs. Compared with a previous study [30], which showed high levels of E2 and neutralizing antibodies, this study verified that the C-vac stimulates the production of a number of antibodies (E2, E0, and NS3 antibodies detected in this study), while the E2-vac stimulates only the E2-specific antibody, verifying that the E2-vac allows DIVA. Additionally, this study showed the E2-vac group pigs developed E2 antibody responses earlier and stronger than the C-vac group pigs (Figure 2A and Figure 3A). Furthermore, this study not only showed that two-dose vaccinations of E2 and C provide protection to pigs against CSFV, it also showed that one-dose vaccinations provide the same protection. It is hard to avoid missing injections or other special situations in clinical practice. Hence, effective one-dose vaccination is useful in clinical practice.

Studies about the efficacy of the classical swine fever live attenuated vaccine and E2 subunit vaccine have one common finding, that is, the neutralizing antibody (NA) induced by vaccines is a good indicator and has a good correlation with the observed protection. In this study, we evaluated antibody titers with an IDEXX ELISA kit as the data showed that the titer of the IDEXX ELISA kit is correlated with NA titer (these data are from a paper in Chinese). We believe that it is feasible to demonstrate the relative neutralizing antibody level or the relative protection level by IDEXX ELISA antibody level alone.

However, the C-vac strain is able to provide clinical protection as early as 3 days post vaccination [31]. Additionally, a previous study reported that another European E2 vaccine provided clinical protection at 14 dpv [32]; however, the onset of protection of the Chinese E2 vaccine was not explored herein. In addition, the animals in this study were challenged with virus on 12 dpv, with a robust antibody response. However, the T cell-medicated immune response is important for evaluating the application of an emergency vaccination strategy. Previous studies showed that animals protected from challenge by vaccination with an E2-based DNA vaccine had increased levels of CSFV-specific IFN-γ-producing cells compared to unvaccinated controls [33]. Additionally, Suradhat et al. showed that CSFV-specific IFN-γ production was detected early after C-strain administration and correlated with protection against CSFV challenge [34]. Other studies were carried out to support this finding. The C-strain was able to induce rapid protection to challenge as early as 5 days post administration and that rapid protection was correlated to induction of CSFV-specific T cell IFN-γ responses [35]. In addition, other studies showed that CD3+ CD4− CD8hi T cell populations were the first and major source of CSFV-specific IFN-γ [36,37], whereas innate immune cells such as NK and gd-T cells were probably not involved in the development of this rapid protection [38]. Therefore, the protection efficiencies between the first vaccination and the first antibody appearance as well as the cell-mediated immune response are significant and need to be explored further.

Infection with virulent CSFV is an immunomodulatory infection that causes immunosuppression [39,40]. In this study, no antibodies were detected in the infection control group pigs from 3 weeks post inoculation (0 dpi to 56 dpi); three animals died, and two were euthanized due to severe clinical symptoms.

Although C-vac has the disadvantage of not allowing DIVA, it is still generally considered a safe and effective vaccine. CSF has been controlled by extensive vaccination with C-vac since the 1950s. Hence, C-vac is still a good choice for farmers. However, CSF was recently demonstrated to occur sporadically or endemically in many regions in China [16,41,42,43,44]. To completely control CSF, an elimination program is the only option. E2-vac is a good candidate licensed vaccine for the control and eradication of CSF, conferring complete protection and allowing DIVA on pig farms. Furthermore, in addition to implementing prophylactic vaccination control measures, more attention must be paid to other measures, such as biosecurity procedures.

Although vaccine manufacturers recommend double vaccination for optimal protection, the effects of both vaccines were evaluated after a single round of vaccination to evaluate the usefulness of both vaccines in an emergency vaccination scenario.

## Figures and Tables

**Table 1:** Numbers of animals with antibody responses after vaccination with one or two doses of C-vac and E2-vac.

Group	Antibody Type	Days Post Vaccination (dpv)																				
		0 *	1	2	5	7	9	12	16	20	23 *	28	30	35^#^	37	40	42	44	47	51	55	56
C-vac two doses	E2	0/5	0/5	0/5	0/5	0/5	0/5	0/5	0/5	1/5	5/5	5/5	5/5	5/5	5/5	5/5	5/5	5/5	5/5	5/5	5/5	5/5
	E0	0/5	0/5	0/5	0/5	0/5	0/5	0/5	0/5	0/5	0/5	0/5	0/5	0/5	0/5	2/5	5/5	5/5	5/5	5/5	5/5	5/5
C-vac one dose	E2	0/5	0/5	0/5	0/5	0/5	0/5	0/5	0/5	0/5	0/5	0/5	0/5	4/5	5/5	5/5	5/5	5/5	5/5	5/5	5/5	5/5
	E0	0/5	0/5	0/5	0/5	0/5	0/5	0/5	0/5	0/5	0/5	0/5	0/5	0/5	0/5	5/5	5/5	5/5	5/5	5/5	5/5	5/5
E2-vac two doses	E2	0/5	0/5	0/5	0/5	0/5	0/5	1/5	5/5	5/5	5/5	5/5	5/5	5/5	5/5	5/5	5/5	5/5	5/5	5/5	5/5	5/5
	E0	0/5	0/5	0/5	0/5	0/5	0/5	0/5	0/5	0/5	0/5	0/5	0/5	0/5	0/5	0/5	0/5	0/5	0/5	0/5	0/5	0/5
E2-vac one dose	E2	0/5	0/5	0/5	0/5	0/5	0/5	0/5	0/5	0/5	0/5	0/5	1/5	5/5	5/5	5/5	5/5	5/5	5/5	5/5	5/5	5/5
	E0	0/5	0/5	0/5	0/5	0/5	0/5	0/5	0/5	0/5	0/5	0/5	0/5	0/5	0/5	0/5	0/5	0/5	0/5	0/5	0/5	0/5

Gray shading: at least one antibody-positive piglet. *: The day with vaccination.

## Supplementary Materials

The following are available online at https://www.mdpi.com/article/10.3390/vetsci8080148/s1, Figure S1: Antibody responses in pigs in the blank control group (DMEM) and infection control group (CSFV). A: ELISA blocking rate of the E2 antibody; B: ELISA blocking rate of the E0 antibody.
