# Supplementary figures and images for: Efficiency Comparison of a Novel E2 Subunit Vaccine and a Classic C-Strain Vaccine against Classical Swine Fever
Source: Vet Sci. 2021 Jul 29;8(8):148. doi: 10.3390/vetsci8080148 (PMC8402791; doi:10.3390/vetsci8080148)

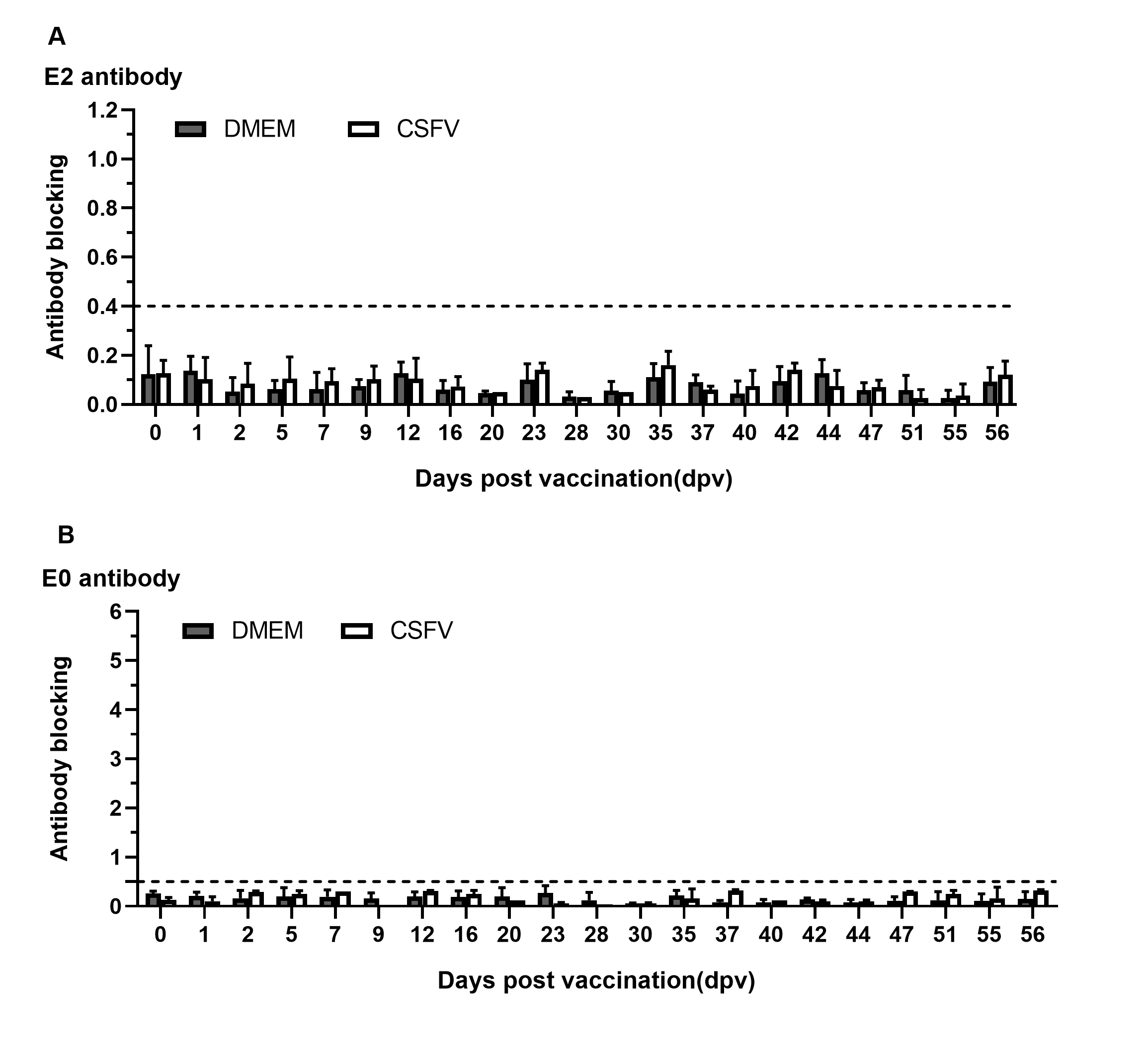

Supplement: Supplementary file 1 [file vetsci-08-00148-s001.zip › vetsci-1308881-supplementary.tif]
